# Supplementary material for: A Critical View of the Application of the APEX Software (Aqueous Photochemistry of Environmentally-Occurring Xenobiotics) to Predict Photoreaction Kinetics in Surface Freshwaters
Source: Molecules. 2019 Dec 18;25(1):9. doi: 10.3390/molecules25010009 (PMC7017383; doi:10.3390/molecules25010009)
Supplement: Supplementary file 1 [file molecules-25-00009-s001.zip › 3.2.4_gcc-4.4.0.zip!/3.2.4_gcc-4.4.0/3.2.4_gcc-4.4.0/doc/octave/html/interpreter/Handling-Warnings.html]

Handling Warnings - Untitled


Previous: Handling Errors,
Up: Errors and Warnings


---

### 12.2 Handling Warnings

Like an error, a warning is issued when something unexpected happens.
Unlike an error, a warning doesn't abort the currently running program.
A simple example of a warning is when a number is divided by zero. In
this case Octave will issue a warning and assign the value `Inf`
to the result.

```
     a = 1/0
          -| warning: division by zero
          => a = Inf
```

- Issuing Warnings- Enabling and Disabling Warnings
